# Analysis of risk factors and development of a predictive model for postoperative delirium in elderly patients undergoing cardiac surgery with general anesthesia

**DOI:** 10.1097/MD.0000000000046021

**Published:** 2025-11-21

**Authors:** Miao-Miao Xu, Jie-Jie Zhou, Meng-Tong Han, Tao Sun, Kang-Li Hui

**Affiliations:** a Department of Anesthesiology, Jinling Hospital, Jinling School of Clinical Medicine, Nanjing University, Nanjing City, Jiangsu Province, China.

**Keywords:** cardiac surgery, elderly patients, nomogram, postoperative delirium, predictive model, risk factors

## Abstract

Postoperative delirium (POD) is a common and serious complication in elderly patients undergoing general anesthesia, particularly with cardiac surgery. Identifying risk factors for POD and developing predictive tools are essential for improving patient outcomes. This retrospective cohort study analyzed data from 238 consecutive elderly patients (aged 60 or older) who underwent elective cardiac surgery at our hospital between January 2021 and December 2024. The study aimed to identify factors contributing to postoperative delirium and to develop a predictive nomogram. Patients were divided into delirium and non-delirium groups based on the occurrence of delirium within 72 hours post-surgery. Statistical analysis was performed using multivariate logistic regression, and a nomogram was developed to predict the risk of POD. Significant factors associated with postoperative delirium included age, Charlson Comorbidity Index, cardiopulmonary bypass, and intraoperative hypotension. The nomogram based on these factors demonstrated strong discriminatory ability with an area under the curve of 0.869 and a sensitivity of 76.21% and specificity of 88.51%. The internal validation showed a high C-index of 0.889, indicating excellent calibration. Decision curve analysis confirmed the model’s clinical utility. The study identified key risk factors for POD in elderly cardiac surgery patients and developed a nomogram with strong discriminatory ability and clinical applicability. This tool could help clinicians identify high-risk patients and improve management to reduce the incidence of POD.

## 1. Introduction

Postoperative delirium (POD) is a common and serious complication in elderly patients following major surgeries, including cardiac surgery. It is characterized by an acute disturbance in attention and awareness, with fluctuating symptoms that can lead to long-term cognitive decline, prolonged hospitalization, and increased mortality. The elderly population, who are often at increased risk due to age-related physiological changes, polypharmacy, and preexisting comorbidities, is particularly susceptible to POD. The incidence of delirium in elderly patients undergoing cardiac surgery is notably high, with estimates ranging from 10% to 50% depending on the patient population and the type of surgery performed.^[1–3]^

Cardiac surgery with general anesthesia introduces several risk factors for delirium, including the nature of the surgery, the use of cardiopulmonary bypass, hemodynamic instability, and the systemic inflammatory response. Moreover, the aging process itself affects the brain’s response to anesthesia and surgical stress, potentially leading to altered neurotransmitter function, reduced cerebral blood flow, and a diminished ability to clear metabolic waste products from the brain. These factors, combined with the complexity of cardiac surgery, create an environment in which elderly patients are vulnerable to the onset of delirium.^[4,5]^ Identifying at-risk patients before surgery is crucial for mitigating the impact of POD and improving patient outcomes. Various risk stratification tools and scoring systems have been developed to predict the likelihood of POD, but there is a need for more refined, individualized models, particularly for patients undergoing high-risk surgeries like cardiac surgery.^[6]^ Predictive modeling have emerged as promising approaches in healthcare to identify complex patterns of risk factors and provide more accurate predictions. This model can incorporate a variety of clinical, demographic, and intraoperative data, potentially offering clinicians valuable insights into which patients are most at risk for POD.^[7,8]^

This study aims to analyze the risk factors for postoperative delirium in elderly patients undergoing cardiac surgery under general anesthesia and to develop a predictive model. By identifying key risk factors and constructing a reliable predictive tool, we hope to improve preoperative risk stratification, guide preventive strategies, and ultimately enhance patient care. Furthermore, understanding the pathophysiology and risk factors associated with delirium in this high-risk population can contribute to the development of more targeted interventions to reduce the incidence and severity of POD.

## 2. Methods

### 2.1. Study design

A retrospective cohort study was conducted at our hospital from January 2021 to December 2024 to analyze the risk factors for postoperative delirium in elderly patients undergoing cardiac surgery with general anesthesia. A total of 238 consecutive patients aged 65 years and older who underwent elective or emergency cardiac surgery were included in the analysis. Based on the presence or absence of delirium within 72 hours postoperatively, patients were divided into 2 groups: the delirium group (n = 28) and the non-delirium group (n = 210). The study adhered to the Strengthening the Reporting of Observational Studies in Epidemiology guidelines, ensuring the research methodology, intent, and protocols were in line with standard practices.^[9]^ Informed consent was obtained from all participants or their legal guardians. The study was reviewed and approved by the ethics committee of our hospital, adhering to the relevant guidelines and the ethical principles of the Declaration of Helsinki. Data confidentiality was maintained, with personal identifiers removed prior to analysis to protect participant privacy.

### 2.2. Inclusion and exclusion criteria

#### 2.2.1. Inclusion criteria

(1) *Age*: Patients aged 60 years and older, as elderly individuals are at a significantly higher risk of developing postoperative delirium.
(2) *Surgical procedure*: Patients undergoing elective cardiac surgery with general anesthesia, including coronary artery bypass grafting (CABG), valve replacement, or combined cardiac surgeries.
(3) *Postoperative follow-up*: Availability of postoperative monitoring for at least 72 hours, which is necessary to assess the development of postoperative delirium.
(4) *Preoperative cognitive status*: Patients with baseline cognitive assessment to evaluate preoperative cognitive impairment, a known risk factor for delirium.

#### 2.2.2. Exclusion criteria

(1) *Severe neurological disorders*: Patients with preexisting severe neurological disorders such as advanced dementia, Parkinson disease, or significant stroke history that would complicate delirium assessment and interpretation.
(2) *History of delirium*: Patients with a documented history of postoperative delirium or any condition that predisposes them to recurrent delirium, which would interfere with the study’s objective to identify new-onset postoperative delirium.
(3) *Uncontrolled acute medical conditions*: Patients with active, uncontrolled medical conditions such as sepsis, severe liver or renal failure, or acute respiratory failure that may independently influence cognitive function.
(4) *Language barrier*: Patients who are unable to communicate in the language of the study due to a language barrier or hearing impairment, preventing accurate delirium assessments or cognitive screening.

### 2.3. Delirium assessment

Postoperative delirium was assessed twice daily, at 08:00 to 10:00 and 20:00 to 22:00, until delirium was identified within 72 hours. The assessment was performed using the Confusion Assessment Method for the Intensive Care Unit (CAM-ICU) and the Richmond Agitation-Sedation Scale (RASS) to evaluate delirium and sedation levels, respectively. The CAM-ICU is a widely used tool for diagnosing delirium in critically ill patients, and the RASS was used to assess agitation and sedation. To minimize the potential influence of sedative and analgesic medications on delirium assessment, if sedative drugs were used, the assessment was postponed until the patient was fully awake after discontinuation of sedation. If the patient was deeply sedated or unable to be awakened (RASS score of −4 or −5), delirium assessments were discontinued, and the patient was classified as comatose for the purpose of the study. Delirium was considered present if the patient met the criteria for delirium on either of the 2 assessment times within the first 72 hours postoperatively.

All delirium assessments were conducted by a dedicated team of trained anesthesiologists and ICU nurses who had received standardized training in the use of CAM-ICU and RASS prior to the study. To ensure data completeness and consistency, all assessments were double-checked by a supervising investigator, and discrepancies were resolved through consensus discussion. Interobserver variability was minimized through periodic calibration meetings and consistent use of standardized assessment protocols.

### 2.4. Data collection and outcome measures

Preoperative data were collected for all eligible patients, including demographic information such as age, sex, years of education, body mass index, surgical history, anesthesia history, underlying comorbidities, and long-term medication use. The Charlson Comorbidity Index (CCI) was calculated for each patient to assess the burden of comorbid conditions. Depression levels were assessed using the Hospital Anxiety and Depression Scale, with scores ranging from 0 to 42, where higher scores reflect greater severity of anxiety and depression. The Barthel Index, which measures the ability to perform activities of daily living, was used, with scores ranging from 0 to 100, where higher scores indicate better functional independence. The European System for Cardiac Operative Risk Evaluation (EuroSCORE) was also recorded to assess perioperative risk. Intraoperative data collected included the type of surgery performed, the duration of anesthesia and surgery, whether cardiopulmonary bypass was used, the occurrence of intraoperative hypotension, and whether bradycardia was observed. Postoperative data included the occurrence of delirium within 72 hours post-surgery, the length of ICU stay, and the 28-day postoperative outcomes, including mortality and discharge status. Intraoperative hypotension was defined as a mean arterial pressure < 65 mm Hg sustained for at least 5 minutes, in accordance with current perioperative guidelines. Bradycardia was defined as a heart rate <50 beats per minute lasting ≥3 minutes or requiring pharmacologic intervention such as atropine administration or adjustment of anesthetic depth.

### 2.5. Statistical analysis

Statistical analysis was performed using SPSS version 26.0 (IBM Corp., Armonk) and R language version 3.6.3 (R Foundation for Statistical Computing, Vienna, Austria). Continuous variables with a normal distribution were expressed as means ± standard deviations ($\bar{x}$_</mathgraphic>_ ± SD), and comparisons between 2 groups were conducted using the independent *t* test. For variables with a skewed distribution, data were presented as medians with interquartile ranges (M[P25, P75]), and group comparisons were made using the Mann–Whitney *U* test. Categorical variables were presented as percentages, with intergroup differences assessed using the Chi-square test. Continuous variables, including age, CCI, EuroSCORE, anesthesia duration, and surgical duration, were treated as continuous variables in the regression analysis to preserve statistical power and avoid information loss. The linearity of these continuous variables with the logit of the outcome was examined before model inclusion. To identify independent risk factors for postoperative delirium, multivariate logistic regression analysis was performed. An events-per-variable (EPV) analysis was conducted to assess the adequacy of the sample size for multivariate logistic regression modeling. The rms package in R was used to develop a nomogram for predicting the risk of postoperative delirium. Receiver operating characteristic curves were plotted, and the consistency index (C-index) was calculated to evaluate the model’s discriminatory ability. The optimal cutoff value for the model was determined using the Youden index method, which provided sensitivity and specificity for the prediction model. The calibration ability of the model was assessed using the Hosmer–Lemeshow goodness-of-fit test. Internal validation was conducted using a bootstrap resampling method (n = 1000 iterations) to calculate the adjusted C-index. Calibration curves were also plotted to visualize the model’s performance in terms of prediction accuracy. Finally, decision curve analysis (DCA) was used to assess the clinical utility of the model. Statistical significance was defined as a *P*-value < .05.

## 3. Results

### 3.1. Comparison of clinical characteristics and outcomes between delirium and non-delirium groups

A comparison of clinical characteristics between the delirium and non-delirium groups is summarized in Table 1. Significant differences were observed in age, CCI scores, EuroSCORE, cardiopulmonary bypass, intraoperative hypotension, intraoperative bradycardia, anesthesia duration, surgical duration, postoperative ICU stay, and 28-day mortality. Patients in the delirium group were significantly older than those in the non-delirium group (71.24 ± 6.15 years vs 66.23 ± 5.45 years, *P* < .001). Additionally, the delirium group had higher CCI scores (1.00 [1.00–2.00] vs 0.00 [0.00–1.00], *P* < .001) and EuroSCORE (5.80 ± 2.75 vs 3.50 ± 2.30, *P* < .001). The proportion of patients requiring cardiopulmonary bypass was significantly higher in the delirium group (78.57% vs 42.38%, *P* < .001). Furthermore, intraoperative hypotension (50.00% vs 7.14%, *P* < .001) and bradycardia (17.86% vs 2.86%, *P* < .001) were more common in the delirium group. Preinduction hemodynamic parameters were comparable: median heart rate before induction was 74 bpm (68–78) versus 73 bpm (67–79) (*P* = .672), and median mean arterial pressure was 91 mm Hg (86–95) vs 90 mm Hg [85–94] (*P* = .558). The median estimated blood loss did not differ significantly (450 mL [320–620] vs 400 mL [280–580], *P* = .121), nor did the rate of intraoperative complications (21.4% vs 15.2%, *P* = .432). The anesthesia duration was significantly longer in the delirium group (5.40 [3.88–7.95] hours vs 4.16 [3.59–4.87] hours, *P* = .017), and surgical duration was extended (3.68 [2.58–6.75] hours vs 3.11 [2.60–3.75] hours, *P* < .001). Postoperative ICU stay was longer (4.76 [2.00–5.75] days vs 2.01 [1.10–3.00] days, *P* < .001), and 28-day mortality higher (17.86% vs 4.76%, *P* = .007). No significant between-group differences were noted in gender, body mass index, surgical history, long-term medication use, Hospital Anxiety and Depression Scale, Barthel Index scores, or surgical type (CABG vs valve surgery) (Table 1).

**Table 1:** Comparison of clinical characteristics between delirium and non-delirium groups.

Item	Delirium group (n = 28)	Non-delirium group (n = 210)	Statistics (*t*/*χ*^2^/*Z*)	*P*-value
Male (%)	17 (60.71)	146 (69.52)	0.888	.346
Age ($\bar{x}$_</mathgraphic>_ ± SD, yr)	71.24 ± 6.15	66.23 ± 5.45	4.499	<.001
BMI ($\bar{x}$_</mathgraphic>_ ± SD)	24.45 ± 2.57	25.06 ± 3.12	0.990	.323
Surgical history (%)	9 (32.14)	90 (42.86)	1.167	.280
General anesthesia history (%)	7 (25.00)	39 (18.57)	0.655	.418
Preoperative long-term medications				
Statins (%)	14 (50.00)	118 (56.19)	0.383	.536
Calcium channel blockers (%)	5 (17.86)	75 (35.71)	3.530	.060
ACEI/ARB (%)	10 (35.71)	86 (41.00)	0.282	.596
Antiplatelet drugs (%)	16 (57.14)	140 (66.67)	0.992	.319
Beta-blockers (%)	15 (53.57)	108 (51.43)	0.045	.831
Estimated blood loss (mL, M [IQR])	450 [320–620]	400 [280–580]	1.558	.121
Surgical complications (%)	6 (21.4)	32 (15.2)	0.621	.432
Preinduction heart rate (bpm, M [IQR])	74 [68–78]	73 [67–79]	0.424	.672
Preinduction MAP (mm Hg, M [IQR])	91 [86–95]	90 [85–94]	0.585	.558
HAD score (M[P25, P75], points)	11.00 (4.00, 15.00)	9.00 (3.50, 13.00)	0.237	.396
BI score (M[P25, P75], points)	90.00 (85.00, 100.00)	95.00 (90.00, 100.00)	0.569	.515
CCI score (M[P25, P75], points)	1.00 (1.00, 2.00)	0.00 (0.00, 1.00)	5.173	<.001
EuroSCORE ($\bar{x}$_</mathgraphic>_ ± SD, points)	5.80 ± 2.75	3.50 ± 2.30	4.853	<.001
Surgical type			1.265	.081
CABG (%)	7 (25.00)	84 (40.00)		
Valve surgery (%)	18 (64.29)	126 (60.00)		
Other (%)	3 (10.71)	10 (4.76)		
Cardiopulmonary bypass (%)	22 (78.57)	89 (42.38)	13.00	<.001
Intraoperative hypotension (%)	14 (50.00)	15 (7.14)	42.41	<.001
Intraoperative bradycardia (%)	5 (17.86)	6 (2.86)	12.61	<.001
Anesthesia duration (M[P25, P75], hours)	5.40 (3.88, 7.95)	4.16 (3.59, 4.87)	5.736	.017
Surgical duration (M[P25, P75], hours)	3.68 (2.58, 6.75)	3.11 (2.60, 3.75)	6.762	<.001
Postoperative hospital stay (M[P25, P75], days)	11 (6.00, 21.00)	10 (7.00, 13.00)	1.298	.078
Postoperative ICU stay (M[P25, P75], days)	4.76 (2.00, 5.75)	2.01 (1.10, 3.00)	6.986	<.001
28-day mortality	5 (17.86)	10 (4.76)	7.174	.007

BI = Barthel Index, BMI = body mass index, CCI = Charlson Comorbidity Index, EuroSCORE = European System for Cardiac Operative Risk Evaluation, HAD = Hospital Anxiety and Depression Scale, MAP = mean arterial pressure.

### 3.2. Multivariate logistic regression analysis of risk factors for postoperative delirium

The multivariate logistic regression analysis identified several factors associated with postoperative delirium in elderly cardiac surgery patients. Age (β = 0.115, OR = 1.121, 95% CI: 1.005–1.253, *P* = .04), CCI score (β = 0.577, OR = 1.780, 95% CI: 1.158–2.726, *P* = .008), and cardiopulmonary bypass (β = 1.755, OR = 5.779, 95% CI: 1.520–21.212, *P* = .01) were significantly associated with an increased risk of postoperative delirium. Intraoperative hypotension (β = 1.559, OR = 4.764, 95% CI: 1.270–18.048, *P* = .019) also showed a significant association. However, intraoperative bradycardia (β = −1.032, OR = 0.356, 95% CI: 0.078–1.702, *P* = .179), anesthesia duration (β = 0.006, OR = 1.006, 95% CI: 0.988–1.023, *P* = .604), surgical duration (β = 0.005, OR = 1.005, 95% CI: 0.986–1.024, *P* = .649), and EuroSCORE (β = 0.198, OR = 1.219, 95% CI: 0.975–1.526, *P* = .081) were not significantly associated with postoperative delirium (Table 2).

**Table 2:** Multivariate logistic regression analysis of risk factors for postoperative delirium in elderly cardiac surgery patients.

Factors	β value	Standard error value	Wald value	OR value	95% CI for OR	*P*-value
Age	0.115	0.056	4.212	1.121	1.005–1.253	.04
CCI score	0.577	0.221	7.045	1.78	1.158–2.726	.008
Cardiopulmonary bypass	1.755	0.675	7.802	5.779	1.520–21.212	.01
Intraoperative hypotension	1.559	0.668	5.52	4.764	1.270–18.048	.019
Intraoperative bradycardia	−1.032	0.78	1.805	0.356	0.078–1.702	.179
Anesthesia duration	0.006	0.009	0.274	1.006	0.988–1.023	.604
Surgical duration	0.005	0.01	0.206	1.005	0.986–1.024	.649
EuroSCORE	0.198	0.116	3.052	1.219	0.975–1.526	.081

CCI = Charlson Comorbidity Index, CI = confidence interval, EuroSCORE = European System for Cardiac Operative Risk Evaluation, OR = odds ratio, β = regression coefficient.

### 3.3. Development of a nomogram for predicting postoperative delirium in elderly cardiac surgery patients

**Figure 1.**
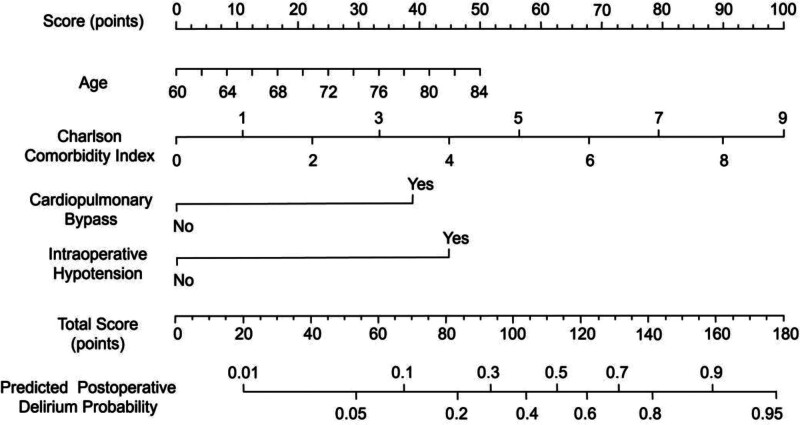
Nomogram for predicting the risk of postoperative delirium in elderly patients undergoing cardiac surgery.

Based on the results from the multivariate logistic regression analysis, 4 significant factors associated with postoperative delirium were identified. A nomogram was developed to predict the likelihood of delirium occurrence after cardiac surgery in elderly patients. The nomogram assigns a score to each factor based on its contribution to the risk of developing postoperative delirium. Each variable is represented on a specific axis of the nomogram, with the corresponding score calculated by drawing a vertical line from the value of the variable to the score scale located above. The total score is obtained by summing the scores for all included variables. This total score is then mapped onto the bottom scale of the nomogram, which corresponds to the predicted probability of the patient developing postoperative delirium. A higher total score indicates a greater risk of delirium (Fig. 1).

### 3.4. Discriminatory ability of the nomogram for predicting postoperative delirium

**Figure 2.**
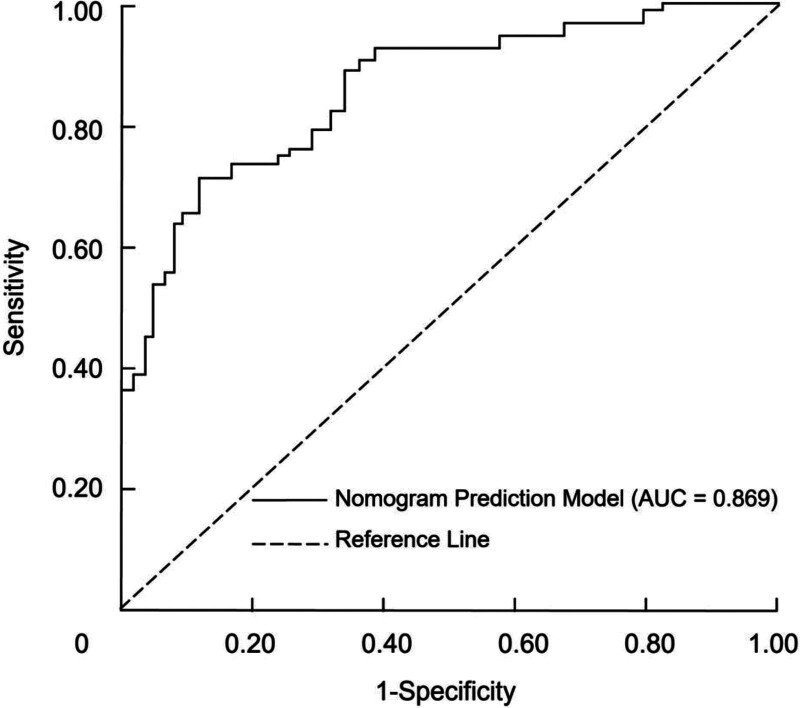
Receiver operating characteristic (ROC) curve for the nomogram in predicting postoperative delirium in elderly cardiac surgery patients.

The discriminatory performance of the nomogram for predicting postoperative delirium risk in elderly cardiac surgery patients was evaluated by using the total risk score as the independent variable and delirium occurrence as the dependent variable. The area under the curve (AUC) for the nomogram model was 0.869 (95% confidence interval, 0.768–0.916), indicating a high level of accuracy in predicting postoperative delirium. At the optimal cutoff point corresponding to the maximum Youden index, the nomogram achieved a sensitivity of 76.21% and a specificity of 88.51% (Fig. 2).

### 3.5. Calibration of the nomogram for predicting postoperative delirium

**Figure 3.**
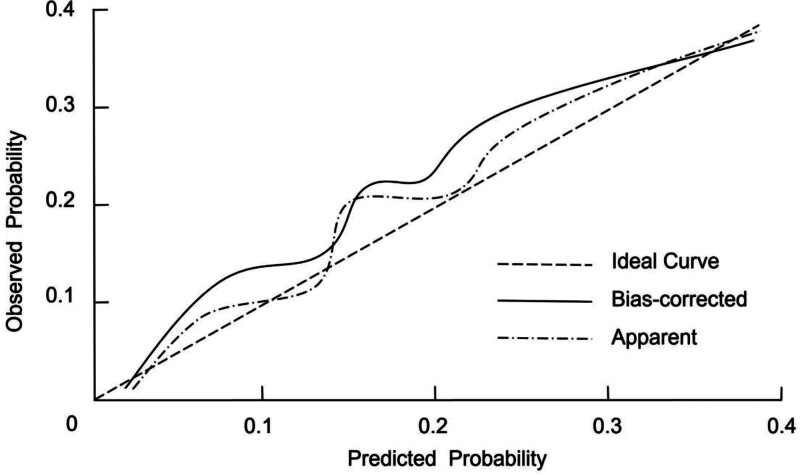
Calibration curve for the nomogram in predicting postoperative delirium in elderly cardiac surgery patients.

To assess the calibration of the nomogram for predicting postoperative delirium, internal validation was performed using the bootstrap resampling method with 1000 iterations. The calibrated C-index was 0.889 (95% CI: 0.836–0.917), indicating a high level of consistency and suggesting that the model provides robust discriminatory ability. Additionally, the Hosmer–Lemeshow goodness-of-fit test yielded a *χ*² value of 2.861 with a *P*-value of .891, indicating that the model’s calibration was excellent. The calibration curve further demonstrated that the predicted probabilities closely matched the observed outcomes, confirming that the model is well-calibrated (Fig. 3).

### 3.6. Clinical utility of the nomogram for predicting postoperative delirium

**Figure 4.**
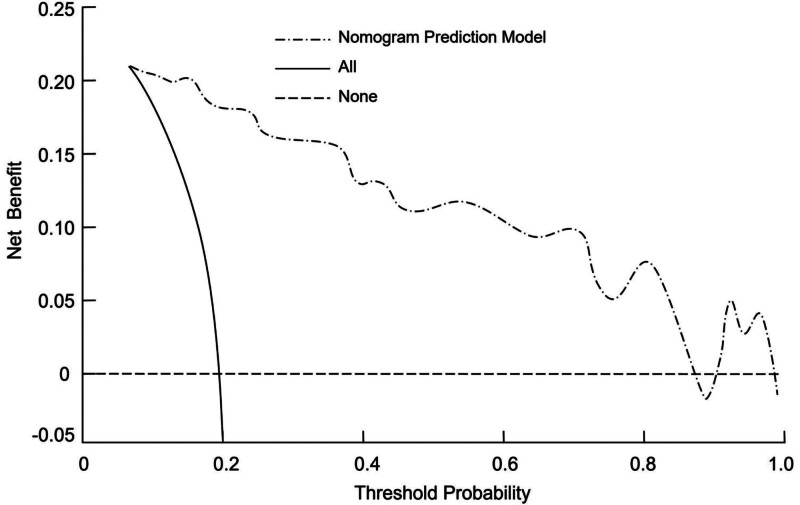
Decision curve analysis (DCA) of the nomogram for predicting postoperative delirium in elderly cardiac surgery patients.

To evaluate the clinical effectiveness of the nomogram for predicting postoperative delirium, DCA was performed. In the DCA curve, the horizontal line represents the scenario where no patients develop postoperative delirium and no interventions are applied, resulting in a net benefit of 0. The diagonal line reflects the scenario in which all patients experience postoperative delirium and receive interventions, with the net benefit showing a negative slope. The net benefit of the nomogram prediction model was significantly higher than both of these extreme scenarios, indicating that the model offers substantial clinical utility (Fig. 4).

### 3.7. Assessment of sample size adequacy for predictive modeling

To evaluate whether the sample size was adequate for logistic regression modeling, an EPV analysis was conducted. In this study, 28 patients developed postoperative delirium, and 4 independent predictors were included in the final multivariate model, resulting in an EPV of 7. Prior studies have shown that an EPV between 5 and 10 still yield reliable models when careful validation methods are used. To enhance robustness, internal validation was performed using 1000 bootstrap resamples, and the model demonstrated excellent discrimination (C-index = 0.889) and calibration, supporting the adequacy of the sample size for preliminary predictive modeling.

## 4. Discussion

POD is a common and serious complication in elderly patients undergoing cardiac surgery, particularly under general anesthesia. The condition is characterized by acute confusion, altered consciousness, and fluctuating mental status, which can lead to prolonged hospital stays, long-term cognitive impairment, and increased mortality. The aging process, combined with the physiological stress of surgery and anesthesia, significantly increases the risk of POD in this vulnerable population. Elderly patients often have multiple comorbidities, cognitive decline, and frailty, all of which contribute to the increased risk of developing delirium. Identifying risk factors for POD is essential for early detection and preventive strategies, which can improve patient outcomes.^[10–12]^

This study identified significant risk factors for postoperative delirium in elderly cardiac surgery patients, including age, CCI, cardiopulmonary bypass, and intraoperative hypotension. A nomogram was developed based on these factors to predict the likelihood of delirium. Compared with previously reported prediction tools or general delirium risk scores, our model provides added value by focusing specifically on elderly patients undergoing cardiac surgery under general anesthesia (a population with distinct perioperative risk profiles). By integrating both preoperative comorbidity burden and key intraoperative hemodynamic parameters, the model offers more individualized risk assessment and greater clinical applicability for perioperative management. The nomogram demonstrated strong discriminatory ability, with an AUC of 0.869 and high sensitivity (76.21%) and specificity (88.51%). Internal validation confirmed its robustness with a C-index of 0.889. Additionally, decision curve analysis showed the model’s substantial clinical utility, indicating its potential for guiding risk stratification and targeted interventions in clinical practice.

Age was found to be a significant predictor of postoperative delirium in our cohort, with older patients having an increased likelihood of developing delirium. This aligns with previous studies suggesting that aging is associated with several pathophysiological changes that may predispose elderly individuals to postoperative complications such as delirium. The aging brain undergoes structural and functional alterations that may impair its ability to respond to the stress of surgery and anesthesia. Additionally, older patients often have more preexisting health conditions, which can contribute to frailty and vulnerability during the postoperative period.^[13,14]^ The CCI, which quantifies the burden of comorbidities, was also significantly associated with postoperative delirium. Patients with higher CCI scores were at greater risk for delirium, reflecting the cumulative effect of multiple chronic conditions, such as hypertension, diabetes, and cardiovascular diseases, which are common in elderly patients undergoing cardiac surgery. The association between comorbidity burden and delirium is well-documented, as underlying health conditions can alter brain function, affect medication metabolism, and interfere with recovery processes, making it more difficult for patients to recover without complications.^[15,16]^

The use of cardiopulmonary bypass (CPB) was another key factor associated with delirium. Our analysis revealed that patients undergoing CPB were more likely to develop delirium, with an odds ratio of 5.779. This is consistent with prior studies, which have suggested that CPB can lead to neuroinflammatory responses, microemboli, and cerebral hypoperfusion, all of which can contribute to postoperative cognitive dysfunction, including delirium. The duration of CPB and the potential for embolic events during surgery may lead to transient ischemic changes in the brain, increasing the risk of postoperative delirium in these patients. Intraoperative hypotension also emerged as a significant risk factor for delirium.^[17,18]^ Hypotension during surgery can reduce cerebral perfusion and oxygenation, which may lead to cognitive dysfunction. Cerebral hypoperfusion has been associated with neuronal injury, inflammation, and the disruption of the blood–brain barrier, all of which could precipitate delirium. The results of this study support the hypothesis that maintaining stable blood pressure during surgery is crucial for preventing postoperative delirium, particularly in elderly patients who may have preexisting cerebrovascular changes.^[19]^

In contrast to the above findings, factors such as intraoperative bradycardia, anesthesia duration, surgical duration, and EuroSCORE were not significantly associated with the development of postoperative delirium in our study. Interestingly, intraoperative bradycardia appeared as a potential protective factor (OR < 1), although this association was not statistically significant. One possible explanation is that mild intraoperative bradycardia may occur in hemodynamically stable patients or during lighter levels of anesthesia, both of which could reduce the stress response and cerebral metabolic demand, thereby providing some degree of neuroprotection. Alternatively, this apparent protective trend may reflect random variation due to the limited number of delirium cases and should therefore be interpreted with caution. Similarly, while longer anesthesia and surgery durations are often cited as contributing to delirium due to prolonged exposure to anesthetic agents, our findings suggest that these factors alone may not be as influential as other variables such as age and comorbidity burden.^[20]^ The EuroSCORE, a well-established risk stratification tool for cardiac surgery, also did not show a significant association with delirium in our analysis. Although it provides a comprehensive assessment of perioperative risk, it may not fully capture the nuances of delirium risk, particularly in elderly patients with complex comorbidities.^[21,22]^

To assist clinicians in identifying patients at high risk for postoperative delirium, we developed a nomogram based on the identified risk factors. The nomogram provides a visual representation of each patient’s individual risk, allowing for personalized prediction of delirium occurrence. This tool may help guide clinical decision-making, enabling healthcare providers to implement preventive measures for high-risk patients. In practical applications, patients identified as high-risk by the nomogram could benefit from preoperative optimization of comorbidities (particularly cardiovascular and metabolic conditions) more stringent intraoperative blood pressure management to maintain cerebral perfusion, and enhanced postoperative monitoring for early detection and intervention. Early mobilization, adequate pain control, and cautious use of sedative medications may further reduce the risk of delirium. The nomogram demonstrated excellent discriminatory ability, with an AUC of 0.869, indicating good accuracy in predicting postoperative delirium. The model’s sensitivity of 76.21% and specificity of 88.51% further support its clinical applicability. Additionally, internal validation using bootstrap resampling provided strong evidence of the model’s robustness, with a high C-index of 0.889, indicating strong calibration and consistent performance. The clinical utility of the nomogram was further validated using DCA, which demonstrated that the model provided a higher net benefit compared to 2 extreme strategies (treating none or treating all patients). This suggests that the nomogram is not only statistically accurate but also practically feasible for guiding individualized perioperative management and improving patient outcomes.

This study has several limitations that should be considered when interpreting the results. First, the study was retrospective in nature, which may introduce inherent biases such as selection bias or incomplete data. Although efforts were made to control for confounding factors, the observational design limits the ability to establish causal relationships between risk factors and postoperative delirium. Second, the study was conducted at a single center, which may restrict the generalizability of the findings to other institutions or populations with different demographic characteristics, surgical practices, or perioperative management strategies. Third, although the EPV analysis suggested that the sample size was generally acceptable, only 28 patients developed postoperative delirium. This relatively small number of events may reduce the statistical power and increase the potential risk of overfitting in the multivariate regression model; therefore, the predictive results should be interpreted with caution. Fourth, only internal validation using bootstrap resampling was performed in this study. While internal validation supports the robustness of the model, external validation in independent cohorts is essential to assess its generalizability and real-world applicability. Fifth, postoperative delirium was assessed only within the first 72 hours after surgery, which might have missed cases occurring later during hospitalization or recovery. Sixth, this study lacked long-term follow-up, making it impossible to evaluate the lasting impact of postoperative delirium on cognitive function and quality of life. Future research should therefore focus on multicenter, prospective studies with larger sample sizes to externally validate and refine the nomogram. In addition, investigating the underlying mechanisms of postoperative delirium (such as neuroinflammation, cerebral hypoperfusion, and vascular changes) could provide valuable insights for targeted prevention and treatment. Furthermore, integrating advanced modeling techniques, including machine-learning approaches, with larger and more diverse datasets may further enhance the model’s predictive accuracy and clinical utility.

## 5. Conclusions

This study identifies key risk factors for postoperative delirium in elderly cardiac surgery patients, including age, comorbidity burden, cardiopulmonary bypass, and intraoperative hypotension. The developed nomogram demonstrated strong discriminatory ability and clinical utility for predicting delirium risk. This tool could aid clinicians in identifying high-risk patients and implementing targeted interventions to improve patient outcomes and reduce the incidence of postoperative delirium.

## Acknowledgments

We would like to express our sincere gratitude to all individuals and organizations that contributed to this study. Special thanks to the research participants for their valuable contributions.

## Author contributions

**Conceptualization:** Miao-Miao Xu, Jie-Jie Zhou, Meng-Tong Han, Tao Sun, Kang-Li Hui.

**Data curation:** Miao-Miao Xu, Jie-Jie Zhou, Tao Sun, Kang-Li Hui.

**Formal analysis:** Miao-Miao Xu, Jie-Jie Zhou, Meng-Tong Han, Tao Sun, Kang-Li Hui.

**Investigation:** Miao-Miao Xu, Jie-Jie Zhou, Meng-Tong Han, Tao Sun, Kang-Li Hui.

**Methodology:** Miao-Miao Xu, Jie-Jie Zhou, Meng-Tong Han, Tao Sun, Kang-Li Hui.

**Supervision:** Miao-Miao Xu, Meng-Tong Han, Tao Sun, Kang-Li Hui.

**Validation:** Miao-Miao Xu, Jie-Jie Zhou, Meng-Tong Han, Tao Sun, Kang-Li Hui.

**Visualization:** Miao-Miao Xu, Jie-Jie Zhou, Meng-Tong Han, Tao Sun, Kang-Li Hui.

**Writing – original draft:** Miao-Miao Xu, Jie-Jie Zhou, Kang-Li Hui.

**Writing – review & editing:** Miao-Miao Xu, Jie-Jie Zhou, Kang-Li Hui.
